# Divergent outcomes of neoadjuvant therapy for locally advanced small-cell lung cancer: two cases report and literature review

**DOI:** 10.1007/s12672-025-04218-z

**Published:** 2025-12-07

**Authors:** Dongfang Qiao, Ziqing Xu, Yizhuo Chen, Zhouqi Zhang, Dongrui Feng, Xin Wang, Ming Dong

**Affiliations:** 1 Department of Lung Cancer Surgery, Tianjin Medical University General Hospital, Anshan Road No.154, Heping District, Tianjin, 300052 People’s Republic of China; 2 Tianjin Key Laboratory of Lung Cancer Metastasis and Tumor Microenvironment, Tianjin Lung Cancer Institute, Tianjin Medical University General Hospital, Tianjin, 300052 People’s Republic of China; 3 Jiamusi University, No. 258 Xuefu Street, Jiamusi City, Heilongjiang Province People’s Republic of China; 4 Department of Pediatric Surgery, Tianjin Children’s Hospital (Tianjin University Children’s Hospital, 238 LongYan Road, Tianjin, 300134 People’s Republic of China; 5 Tianjin Health Management and Promotion Institute, Tianjin, People’s Republic of China

**Keywords:** Small cell lung cancer, Neoadjuvant therapy, Immunotherapy, Tumor mutational burden, Gene mutation, PD-L1

## Abstract

This article reports two cases of stage IIIB small cell lung cancer (SCLC) patients who underwent neoadjuvant immunotherapy combined with chemotherapy, resulting in markedly different clinical outcomes, and explores the potential molecular mechanisms behind these differences. Both patients were diagnosed with stage IIIB small cell lung cancer through imageological examination and CT-guided percutaneous lung biopsy. They received three cycles of neoadjuvant treatment with “etoposide + carboplatin + serplulimab” followed by surgical resection of the lesions. Genetic testing for solid tumors was conducted before and after treatment. The results showed that Case 1 exhibited multiple gene mutations, including *FBXW7* and *KRAS*, with a tumor mutational burden (TMB) of 17.65 muts/Mb before treatment. After neoadjuvant therapy, only the *PTEN* mutation remained, and TMB decreased to 0.00 muts/Mb, with no cancer cells observed in the postoperative pathology, leading to an assessment of pathological complete response (pCR). In contrast, Case 2 showed *MET* and *PTEN* mutations and *MYC* gene amplification both before and after treatment, with TMB increasing from 23.72 muts/Mb to 28.13 muts/Mb, resulting in an assessment of disease progression (PD) post-surgery. This study emphasizes the potential value of neoadjuvant therapy in locally advanced SCLC patients and highlights the importance of genetic testing in predicting treatment responses and guiding personalized treatment strategies. Further research is needed to explore more effective therapeutic strategies to improve the prognosis of SCLC patients.

## Introduction

Small-cell lung cancer (SCLC) represents 13–15% of all new lung cancer cases in the United States. This type of tumor is known for its early dissemination, leading to 80–85% of patients being diagnosed with extensive-stage SCLC (ES-SCLC) [1]. Neoadjuvant therapy for SCLC refers to treatment administered prior to surgery, aimed at reducing tumor size, increasing the success rate of surgical intervention, and improving patient prognosis. In recent years, the use of immunotherapy, such as PD-1/PD-L1 inhibitors, has become increasingly common in SCLC, and clinical studies are beginning to explore its potential as a neoadjuvant treatment option [2, 3]. 

Recent studies have indicated that neoadjuvant therapy combined with surgical intervention can improve postoperative survival outcomes. For instance, some research has shown that neoadjuvant treatment increases the rate of pathological complete response (pCR) in lung cancer patients, thereby enhancing long-term survival rates [4, 5]. A recent clinical trial supports the combination of serplulimab with chemotherapy as a first-line treatment option for patients with ES-SCLC [6]. In this report, we present two cases of stage IIIB SCLC patients who received similar neoadjuvant treatment regimens but had markedly different outcomes. Additionally, we performed genetic testing related to SCLC on both patients before and after treatment.

## Case report

### General conditions

**Case1**: A 66-year-old female with 30-pack-year smoking history presented with hemoptysis. The patient underwent abdominal CT, head MRI, and bone scintigraphy, which ruled out systemic metastasis. Chest CT demonstrated a 46 × 43 mm right lower lobe mass (T3) with obstructive pneumonia and enlarged hilar/mediastinal lymph nodes (N2, short-axis 8–12 mm), staging IIIB (T3N2M0). Serum tumor markers were elevated: CEA 21.06 ng/mL, ProGRP 227.18 pg/mL, NSE 35.62 µg/L. Biopsy confirmed small cell carcinoma (diffuse Syn/INSM-1+, Ki-67 95%). Genomic profiling revealed driver mutations: *FBXW7* p.R479* nonsense mutation (VAF 67.70%), *KRAS* p.G12C missense mutation (VAF 34.90%), *PTEN* p.P89Lfs10 frameshift mutation (VAF 30.0%), and *RB1* p.R255 nonsense mutation (VAF 49.10%). Tumor mutational burden was 17.65 muts/Mb.

Treatment with etoposide-carboplatin-serplulimab (3 cycles) induced rapid biomarker normalization: ProGRP decreased from 227.18 to 45.23 pg/mL, CEA from 21.06 to 2.89 ng/mL, and NSE from 35.62 to 12.06 µg/L. Imaging confirmed partial response (>30% reduction) at 2 months. Due to the patient expressed a strong preference for surgery. Upon consultation with a multidisciplinary team, it was considered that the patient’s tumor was potentially resectable. Based on the Checkmate 816 study [7], we believed that performing the surgery at this time might be an optimal strategy. Previous studies have also reported the benefits of this treatment approach for such patients [4, 5]. Re-evaluation with contrast-enhanced CT revealed no significant change in the mediastinal lymph nodes compared to the pre-treatment scan. Therefore, the patient opted for surgical intervention. Subsequent VATS right lower lobectomy achieved pCR: no residual carcinoma in primary site or lymph nodes (stations 2,3,4,7,10,11). Postoperative molecular analysis showed reduced *PTEN* mutation frequency (30%→1.50%), undetectable TMB (17.65 vs. 0 muts/Mb), and PD-L1 negativity (TPS/CPS = 0%).

**Fig. 1:**
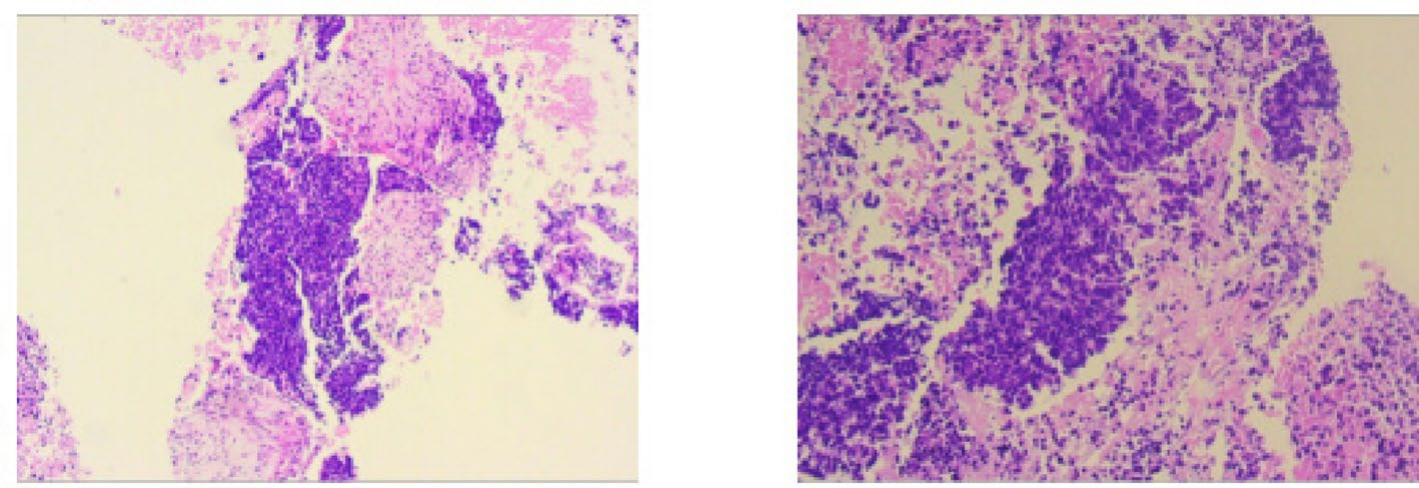
Case1 Histopathological images of percutaneous aspiration biopsy specimens

**Fig. 2:**
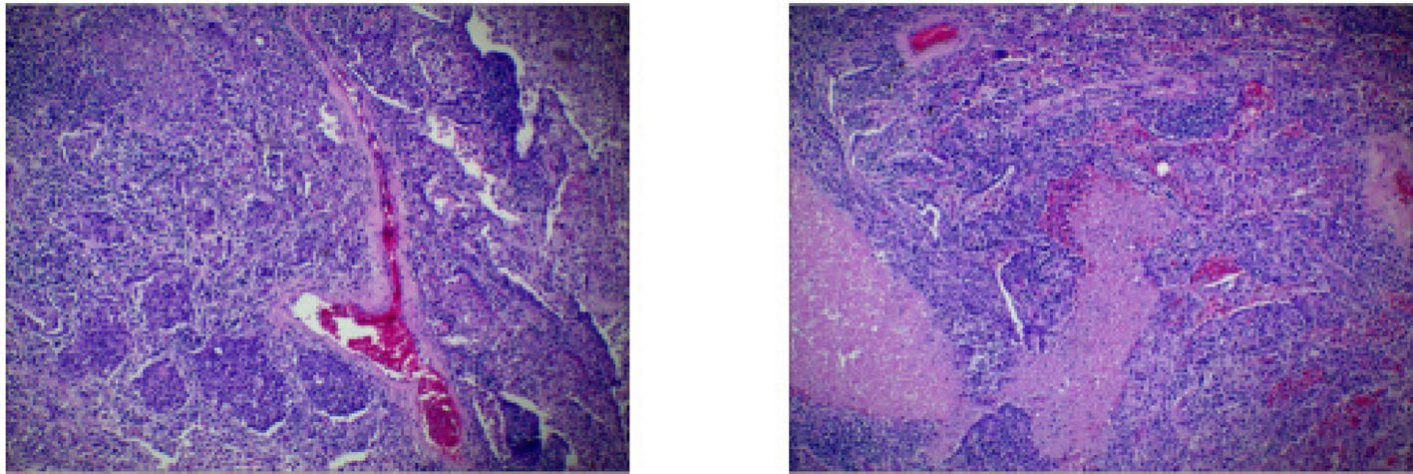
Case1 Histopathological images of surgical specimens

**Fig. 3:**
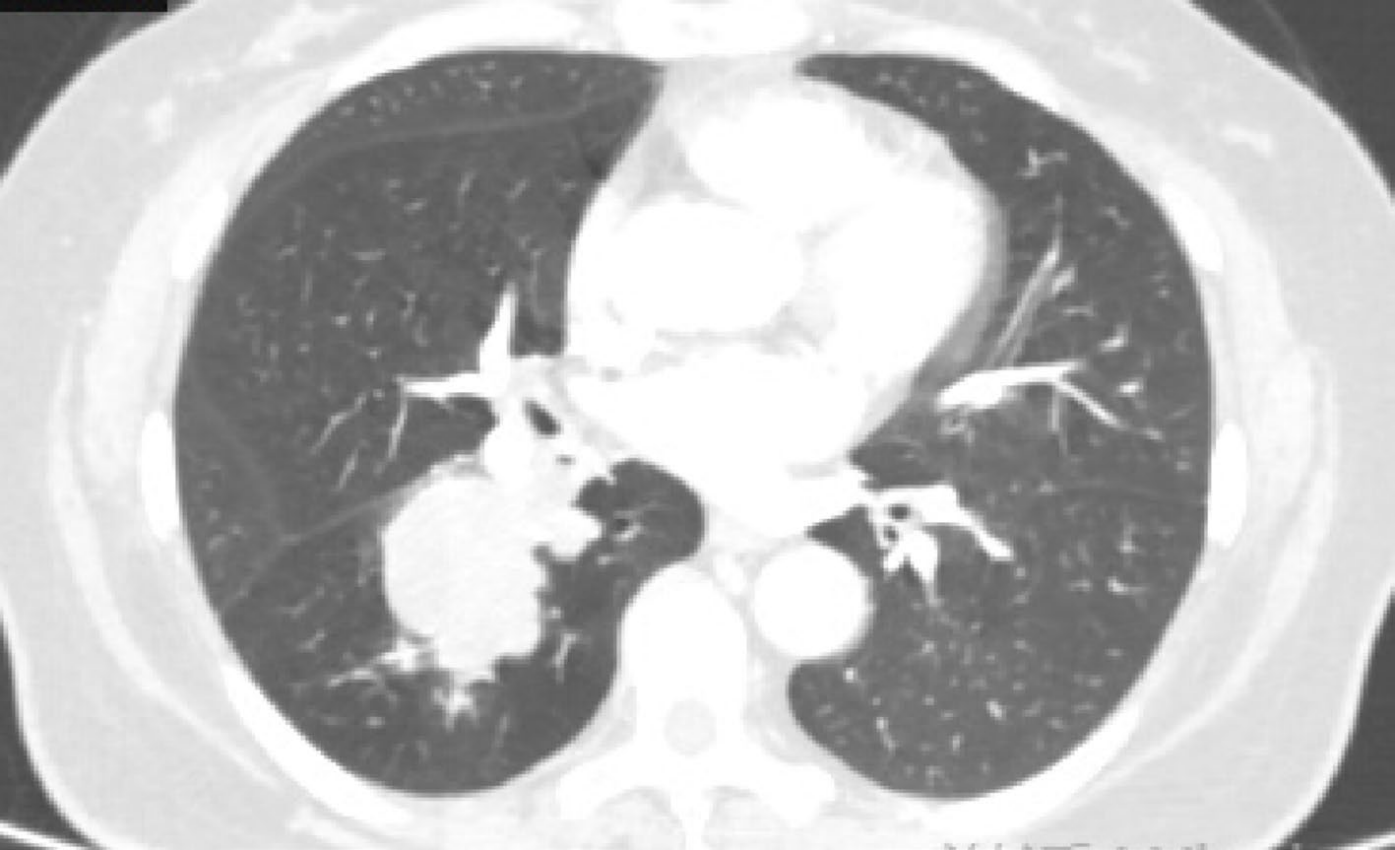
Case1 17 July 2024 CT

**Fig. 4:**
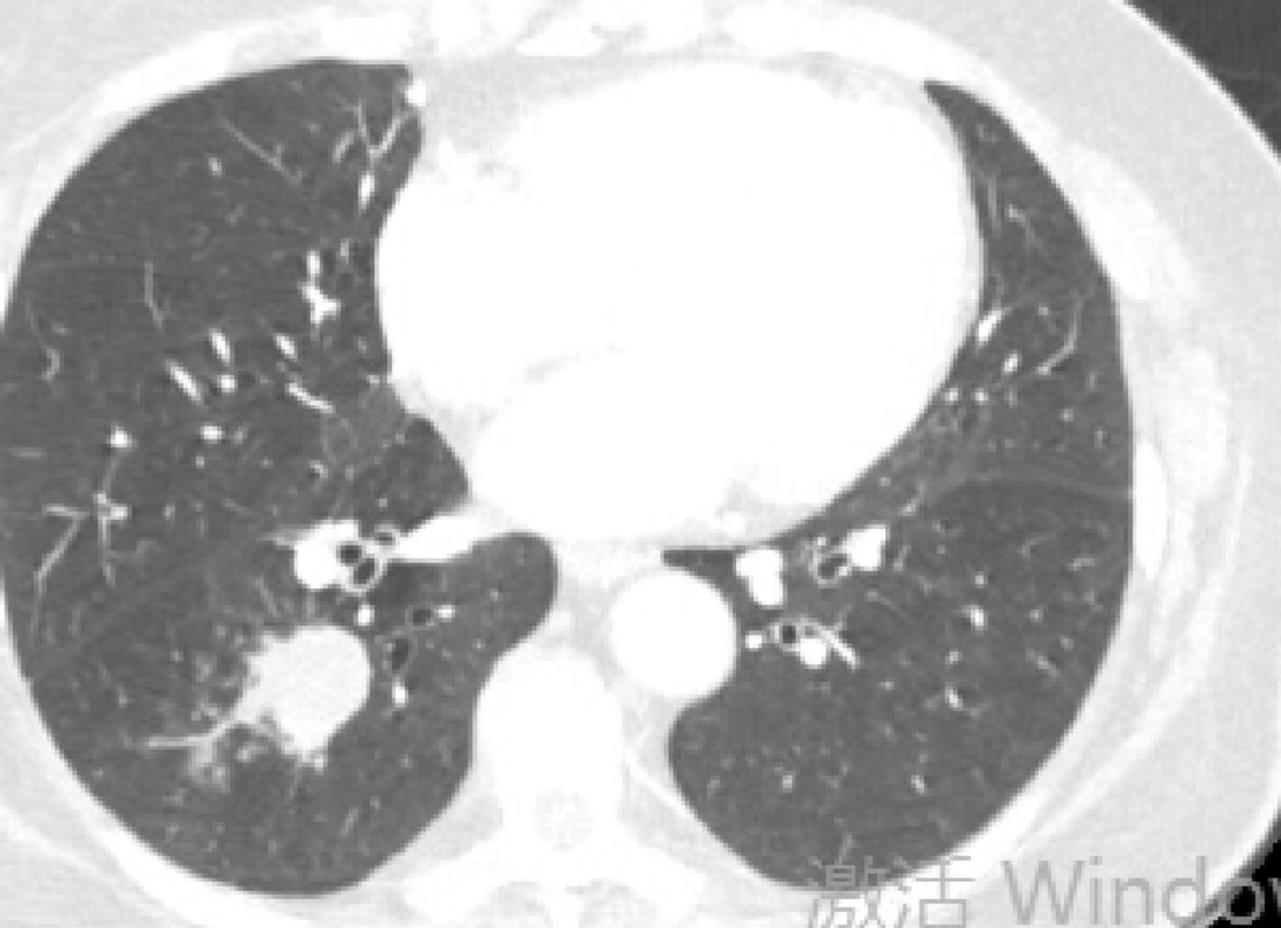
Case1 31 October 2024 CT

**Case2**: A 70-year-old male with 40-pack-year smoking history presented with cough and white sputum. The patient underwent abdominal CT, head MRI, and bone scintigraphy, which ruled out systemic metastasis. Chest CT revealed a lobulated 53 × 41 mm right lower lobe mass with pleural thickening (T4), with enlarged mediastinal lymph nodes, staged as IIIB (T4N2M0). Biopsy confirmed poorly differentiated small cell carcinoma (CD117/BRG1/INI1+, Ki-67 85%). Genomic analysis identified MET chr7:exon2 fusion (VAF 12.15%), PTEN p.T319* nonsense mutation (VAF 57.30%), TP53 p.R175H missense mutation (VAF 61.40%), and MYC amplification (copy number 4.98), with TMB 23.72 muts/Mb.

Treatment with etoposide-carboplatin-serplulimab (3 cycles) induced transient NSE elevation (28.08 µg/L) followed by normalization (12.65 µg/L). Imaging showed partial response (68.3% tumor reduction, 27 mm × 20 mm) after cycle 2, but paradoxical progression (38 mm × 33 mm) post-cycle 3. Contrast-enhanced CT demonstrated no significant alteration in the mediastinal lymph nodes. The tumor was assessed as stable disease (SD), thorough preoperative evaluation indicates that the lesion has been downstaged and is now amenable to surgical intervention. Due to the patient expressed a strong preference for surgery. Upon consultation with a multidisciplinary team, it was considered that the patient’s tumor was potentially resectable. Based on the Checkmate 816 study, [7] we believed that performing the surgery at this time might be an optimal strategy. Previous studies have also reported the benefits of this treatment approach for such patients [4, 5]. Therefore, the patient opted for surgical intervention. Post-progression VATS lobectomy confirmed residual poorly differentiated carcinoma with vascular invasion (CK/TTF-1/CD56/Syn/CgA+). Molecular profiling demonstrated reduced MET fusion (VAF 5.90% vs. 12.15%) and PTEN mutation (42.30% vs. 57.30%), stable TP53 mutation (57.60% vs. 61.40%), decreased MYC amplification (CN 4.48 vs. 4.98), and elevated TMB (28.13 vs. 23.72 muts/Mb). PD-L1 expression increased post-treatment (TPS = 20%, CPS = 30%).

**Fig. 5:**
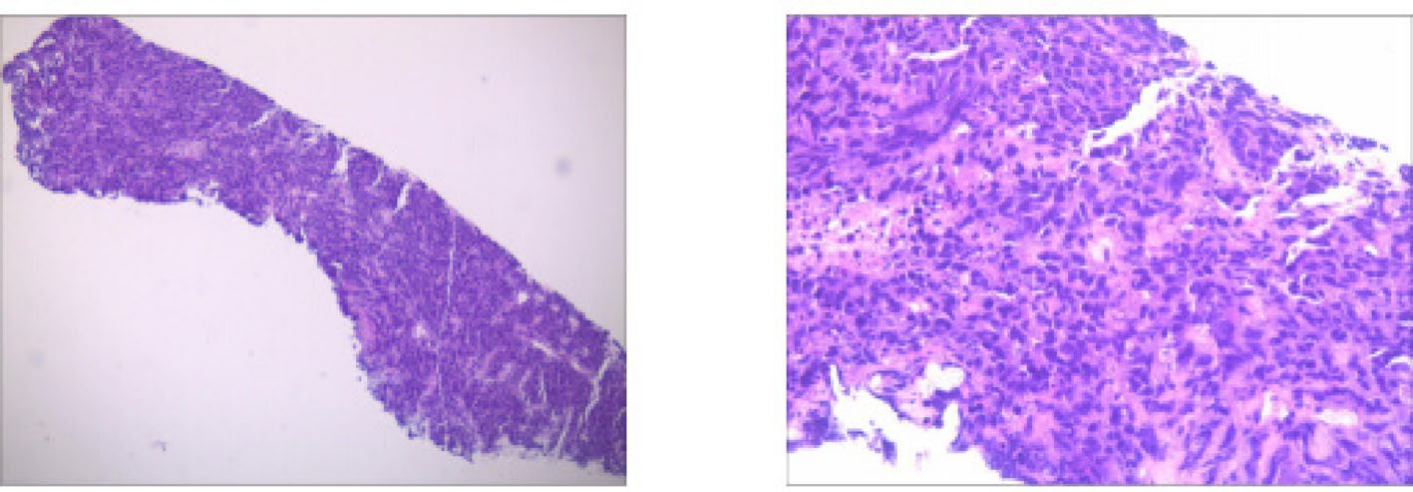
Case2 Histopathological images of percutaneous aspiration biopsy specimens

**Fig. 6:**
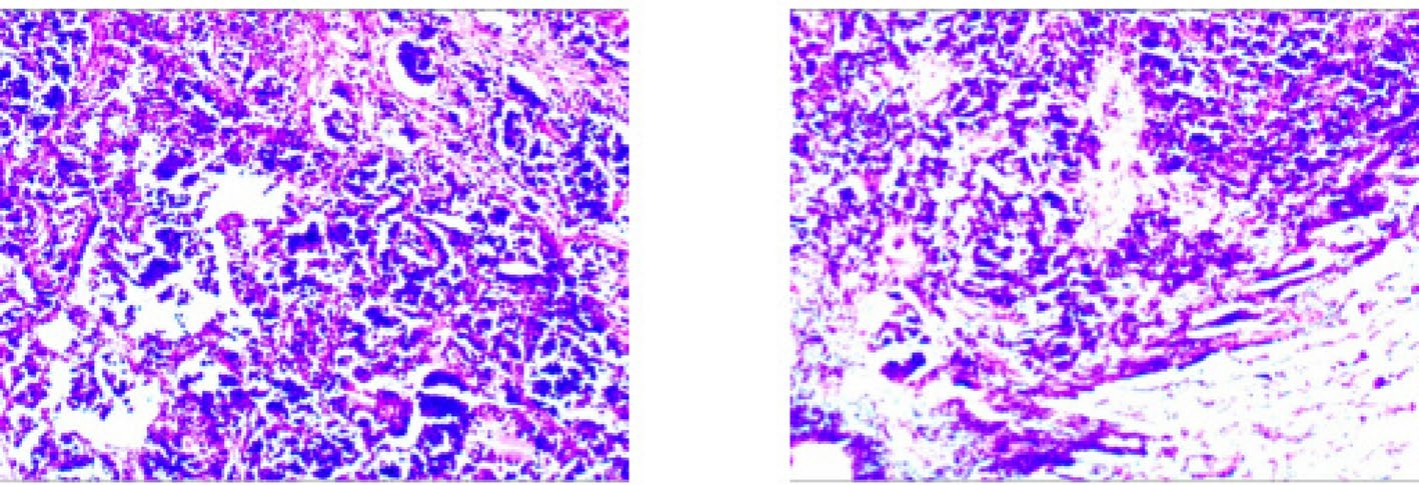
Case2 Histopathological images of surgical specimens

**Fig. 7:**
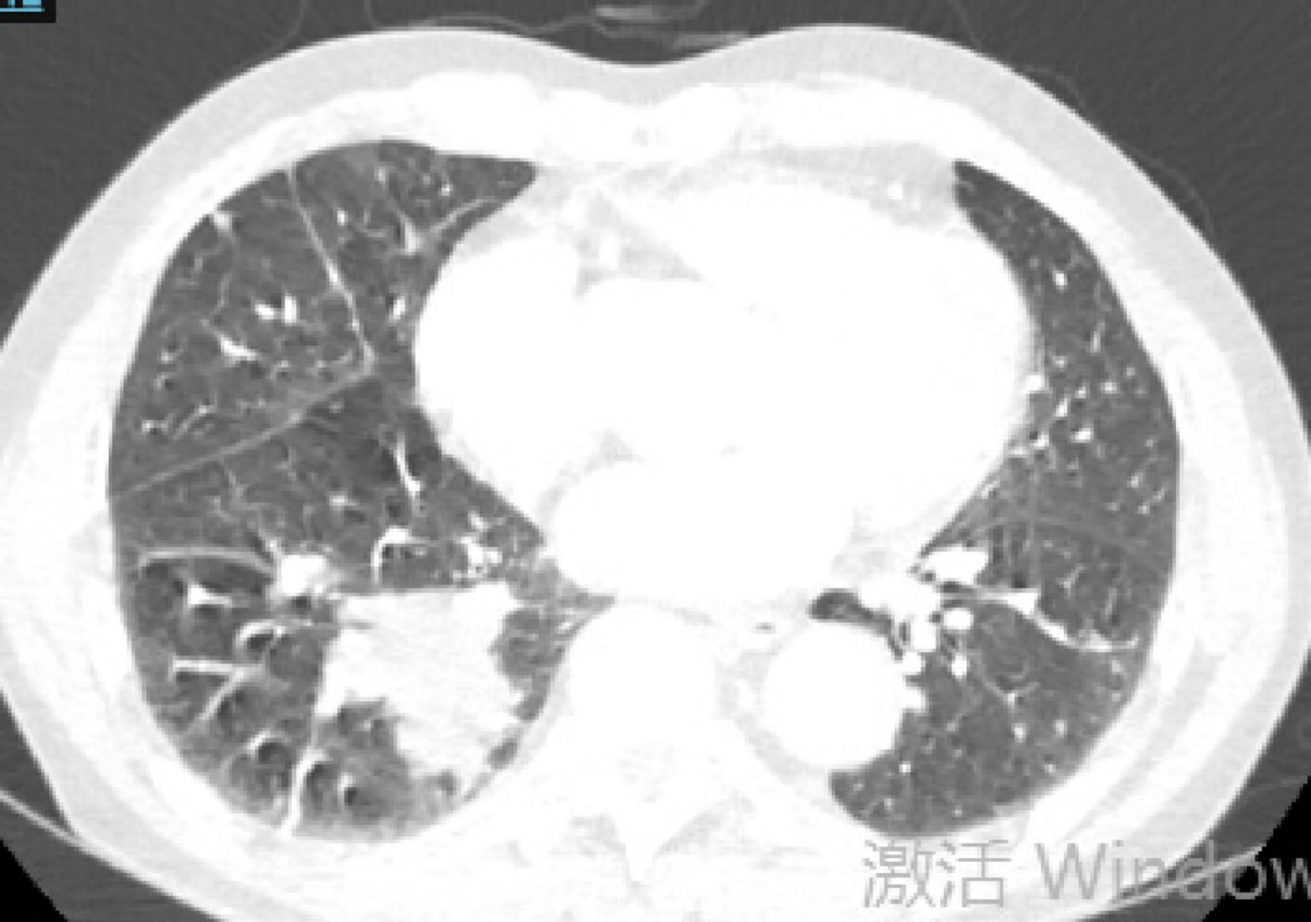
Case2 15 July 2024 CT

**Fig. 8:**
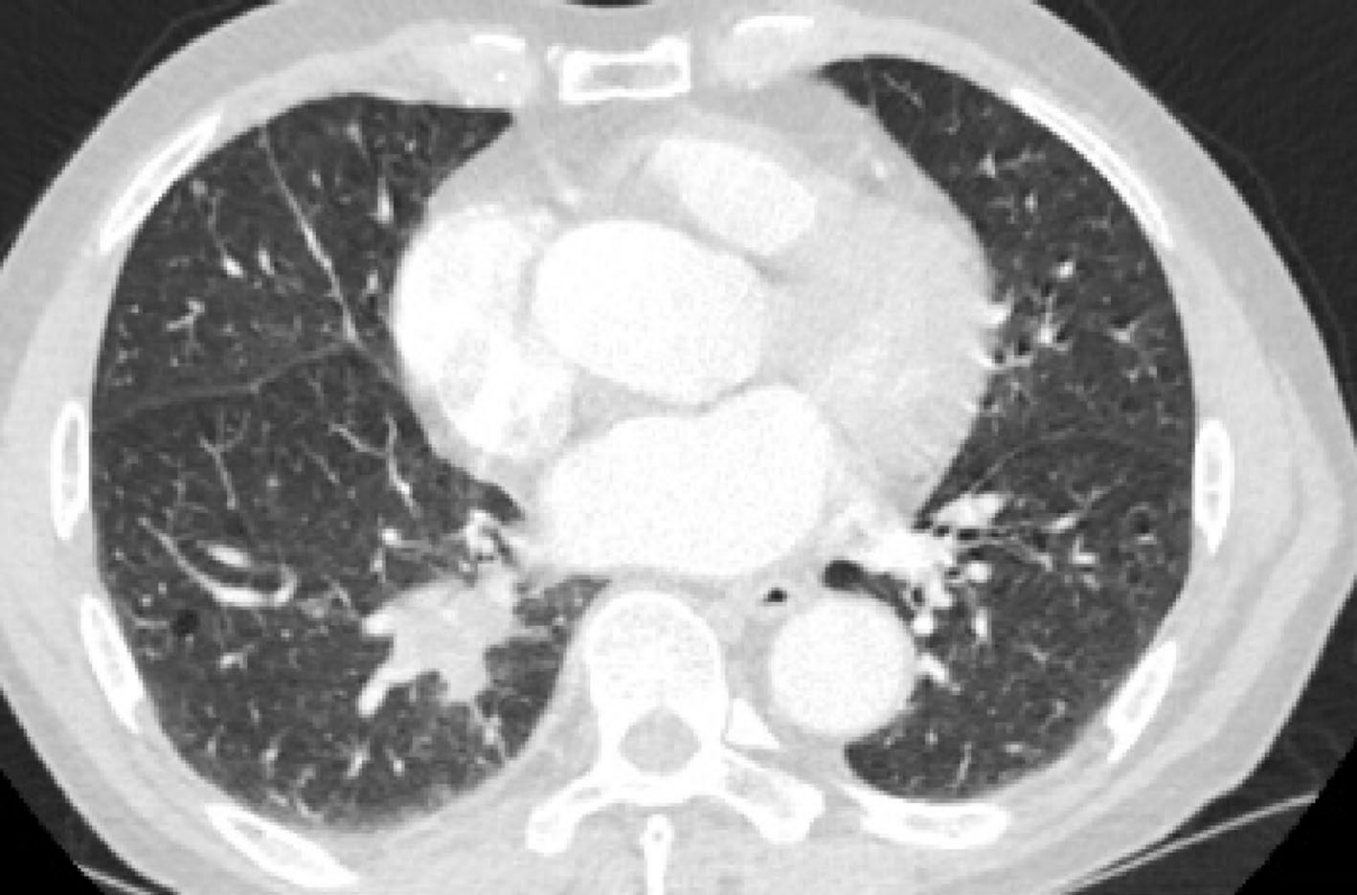
Case2 23 September 2024 CT

**Fig. 9:**
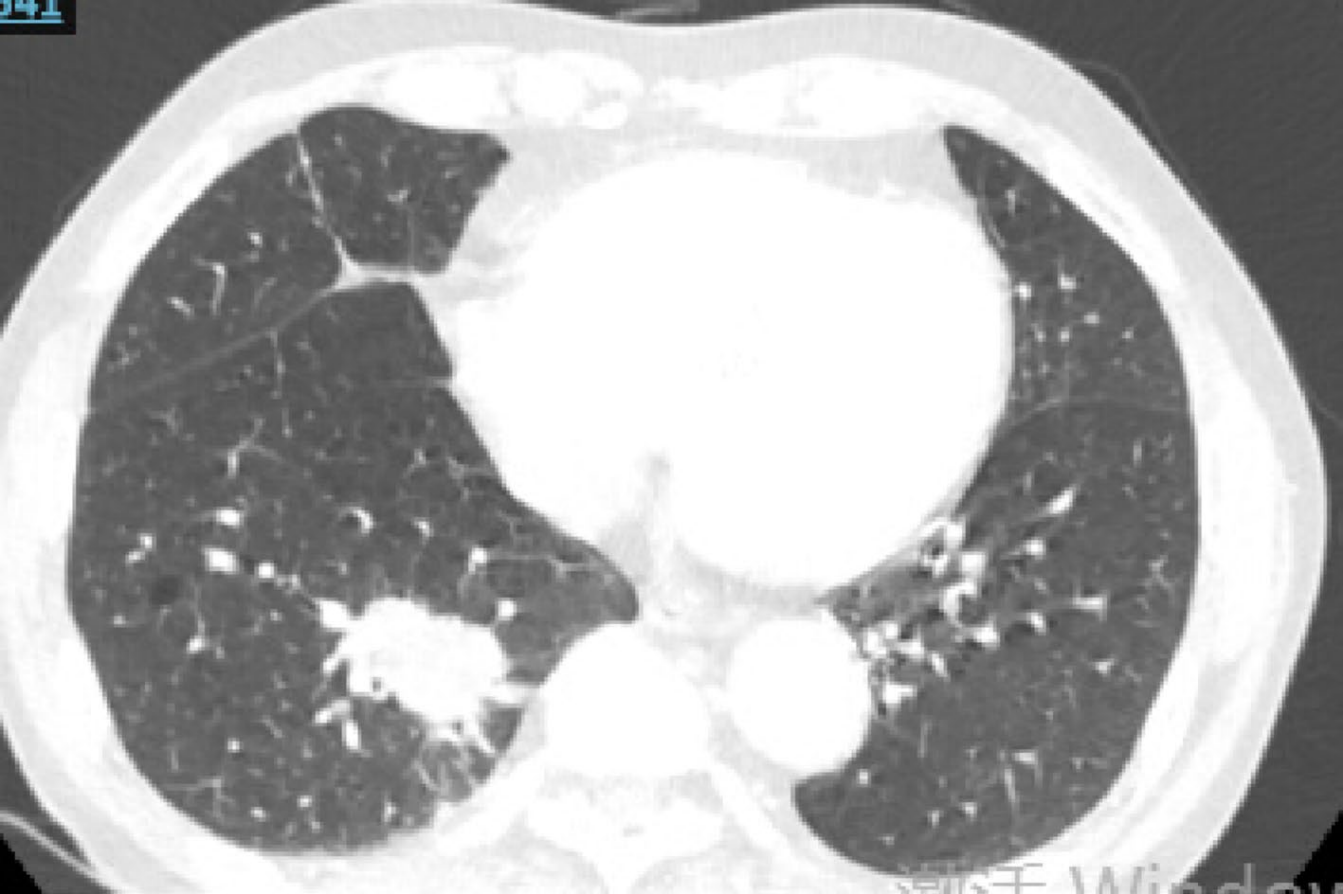
Case2 1 November 2024 CT

## Discussion

This study analyzes two elderly stage IIIB SCLC cases treated with neoadjuvant EC + anti-PD-1 therapy and surgery. Despite identical staging and treatment, genomic profiling revealed divergent driver alterations (MYC amplification, TP53/RB1 mutations, MET fusions) and immune features (PD-L1/TMB dynamics), explaining their opposing outcomes. Unlike non-small cell lung cancer (NSCLC), SCLC lacks molecular subtyping frameworks, highlighting the need for genomic characterization to predict treatment responses [8]. 

The therapeutic management of Case 1 was straightforward and unanimously agreed upon, resulting in a favorable outcome. In contrast, the decision to proceed with surgery in Case 2 was more nuanced and subject to debate, acknowledging that some perspectives consider surgical intervention potentially harmful in such scenarios. However, this decision was deliberate and based on several key considerations. Foremost was the patient’s persistent and fully informed desire for surgical treatment after detailed counseling. This patient preference was evaluated alongside emerging literature suggesting the potential benefit of this approach [4, 5, 7]. A repeat Multidisciplinary Team (MDT) discussion concluded that the radiographic findings did not meet RECIST v1.1 criteria for progressive disease (PD). Specifically, the sum of the target lesions’ longest diameters had increased by less than 20% from the nadir and remained substantially smaller than the pre-treatment baseline, consistent with Stable Disease (SD) [9]. Furthermore, it was posited that the focal growth might represent an atypical response pattern, such as oligoprogression or pseudo-progression, which can occasionally occur with immunotherapy, rather than unequivocal biological progression [10]. The subsequent confirmation of a favorable pathological response post-surgery supported our individualized decision. Nonetheless, this case serves as a critical reminder that surgical candidacy in such complex scenarios requires extremely careful patient selection and prudent judgment.

Comparative genomic profiling of two neoadjuvant therapy cases revealed distinct clonal dynamics. In Case 1, pre-treatment analysis identified four actionable alterations (*FBXW7* p.R479* [VAF67.70%], *KRAS* p.G12C [34.90%], *PTEN* p.P89Lfs10 [30.00%], and *RB1* p.R255 [49.10%]) with baseline TMB 17.65 mutations/Mb. Post-treatment evaluation showed dramatic clonal contraction with only residual *PTEN* mutation (VAF 1.50%), complete TMB clearance (0.00/Mb), and negative PD-L1 expression (TPS/CPS = 0). In contrast, Case 2 demonstrated persistent oncogenic drivers through therapy: *MET* chr7:exon 2 fusion (VAF 12.15%→5.90%), *PTEN* p.T319* (57.30%→42.30%), *TP53* p.R175H (61.40%→57.60%), and *MYC* amplification (CN 4.98→4.48), accompanied by paradoxical TMB elevation (23.72→28.13/Mb). This molecular divergence likely reflects intratumoral heterogeneity and spatial-temporal sampling biases, where distinct subclones with differential mutation burdens were selectively captured in sequential biopsies.

Interestingly, we identified a KRAS mutation in Case 1.Genomic studies by George et al. and Rudin et al. have unequivocally demonstrated that KRAS mutations are notably absent in small cell lung cancer (SCLC). In contrast to lung adenocarcinoma, SCLC pathogenesis is predominantly driven by the near-universal inactivation of tumor suppressors TP53 and RB1, with KRAS playing no significant role in its oncogenesis [11]. In a pivotal phase III clinical trial investigating first-line immunotherapy combined with chemotherapy for extensive-stage SCLC, the KRAS mutation status was neither reported nor assessed for its impact on therapeutic efficacy. This conspicuous absence further substantiates that KRAS mutations are exceedingly rare in SCLC and are not considered a relevant factor in its clinical management or prognostic evaluation [12]. The cooperative inactivation of TP53 and RB1 constitutes a fundamental genetic hallmark of small cell lung cancer (SCLC). Beyond their established role in pathogenesis, emerging evidence indicates that RB1 status serves as a predictive biomarker for chemotherapy response. Specifically, patients with RB1-wild-type SCLC exhibit inferior responses to standard chemotherapy and suffer from shorter survival. This underscores the clinical imperative to explore alternative strategies, such as early intervention with targeted therapies or immunotherapy, for this distinct molecular subset [8, 18]. Furthermore, studies by Liu et al. highlight that many p53 mutants not only lose their tumor-suppressive functions but also acquire potent oncogenic gain-of-function (GOF) properties, which drive tumor progression and therapy resistance. In the context of small cell lung cancer (SCLC), these TP53 GOF mutations contribute to poor treatment efficacy by fostering genomic instability, enhancing migratory and invasive capacities, and conferring multi-faceted therapy resistance [20–22]. Here, we reviewed the literature and summarized the mechanisms or significance of mutations of the relevant genes.

**Table:** 

Gene	Mechanism and key findings	Literature
*FBXW7*	*FBXW7*, the substrate-recognition subunit of the SCF ubiquitin ligase complex, targets proto-oncoproteins (e.g., cyclin E, c-MYC, mTOR) for ubiquitination and proteasomal degradation. Specifically, *FBXW7*-mediated mTOR degradation suppresses mTOR signaling. Its genetic inactivation hyperactivates this pathway, driving tumorigenesis via sustained oncogenic signaling.	[13]
*KRAS*	In cancers, hotspot mutations (codons 12/13/61) in *HRAS*,* KRAS*, or *NRAS* disrupt GTP cycling, trapping RAS in a persistently active GTP-bound state resistant to GAP regulation. This hyperactivates downstream pathways—notably MAPK (RAS-RAF-MEK-ERK) and PI3K-AKT-mTOR—to drive tumor growth by enhancing proliferation, survival, and metastasis.	[14, 15]
*PTEN*	The *PTEN* tumor suppressor gene, via its lipid phosphatase activity, inhibits PI3K/AKT/mTOR signaling by dephosphorylating PIP3. Truncating mutations cause loss of function, leading to constitutive PI3K pathway activation and oncogenesis.	[16]
*RB1*	In SCLC, *RB1* mutations are common and linked to better chemotherapy response. The *RB1*-encoded retinoblastoma protein (RB) suppresses tumor growth by binding E2F transcription factors to silence proliferation genes, regulating cell cycle checkpoints, apoptosis, and differentiation. *RB1* dysfunction disrupts these processes, driving malignancy.	[8, 17, 18]
*MET*	The *MET* proto-oncogene encodes the c-MET receptor tyrosine kinase, which binds HGF and activates downstream pathways. Genetic alterations (mutations, amplifications, etc.) or c-MET overexpression drive aberrant MET/HGF signaling, promoting tumor initiation, metastasis, and drug resistance across cancers.	[19]
*TP53*	The *TP53* tumor suppressor gene encodes p53, a key regulator of genomic stability and cell proliferation control. *TP53* mutations impair its ability to trigger stress responses, cell cycle arrest, or apoptosis. The p53-p.Arg175His (R175H) hotspot mutation disrupts its DNA-binding domain, reducing tumor-suppressive functions and promoting oncogenesis. This mutation weakens activation of *p53* target genes, induces apoptosis resistance, and grants gain-of-function properties that enhance aberrant transcription and cell migration.	[20–22]
*MYC*	The *MYC* proto-oncogene regulates cell proliferation, differentiation, and apoptosis. Its overexpression drives tumor development. *MYC* amplification correlates with poor prognosis in early-stage lung adenocarcinoma and is observed in 6%–25% of SCLC cases, linked to reduced survival and therapy resistance.	[23–25]

TMB is defined as the total number of somatic mutations (including substitutions, insertions, and deletions) per megabase (Mb) within the protein-coding exonic regions of a tumor genome [26] Mechanistically, tumors with elevated TMB harbor increased neoantigen loads, enhancing immunogenic visibility and T-cell-mediated antitumor responses. Clinical studies validate TMB as a predictive biomarker for immune checkpoint inhibitor (ICI) efficacy, where higher TMB correlates with improved objective response rates and progression-free survival due to enhanced immune recognition of neoantigen-expressing tumor cells [27]. Despite exhibiting a higher baseline TMB compared to Case 1, Case 2 demonstrated paradoxically inferior therapeutic outcomes following chemoimmunotherapy. This observation challenges the presumed correlation between TMB levels and immunotherapy efficacy in SCLC, suggesting that either: TMB may lack prognostic/predictive value in SCLC, uncharacterized genomic variants with clinical significance (particularly the 44 variants of unknown significance identified in Case 2) may modulate treatment responsiveness, or complex tumor-immune microenvironment interactions override TMB-driven immunogenicity. Notably, while SCLC tumors with TMB >10 mutations/Mb may exhibit sensitivity to immune checkpoint inhibitors (PD-1/PD-L1 inhibitors), elevated TMB does not invariably predict enhanced therapeutic efficacy. These findings highlight the need for systematic investigations integrating multi-omics profiling to delineate the interplay between somatic mutations, clonal architecture, and immune evasion mechanisms in SCLC [26]. Although some studies suggest that patients with high TMB may exhibit better responses to immunotherapy, therapeutic efficacy is concurrently influenced by factors such as the tumor microenvironment, immune evasion mechanisms, tumor-infiltrating lymphocytes (TILs), and PD-L1 expression. Therefore, predicting the efficacy of immunotherapy in SCLC requires a comprehensive evaluation of multiple biomarkers and individual clinical characteristics. Furthermore, findings from the KEYNOTE-042 and KEYNOTE-024 trials demonstrated that PD-1 inhibitors (e.g., pembrolizumab) show differential efficacy in advanced NSCLC patients depending on PD-L1 expression levels. Higher PD-L1 expression correlates with improved treatment sensitivity, prolonged progression-free survival (PFS) and overall survival (OS), and reduced incidence of treatment-related adverse events [28, 29]. Notably, our study presents a paradoxical clinical observation: Case 2 with higher PD-L1 expression (TPS = 20%, CPS = 30) exhibited diminished sensitivity to PD-1 inhibitor therapy compared to Case 1 demonstrating lower PD-L1 levels (TPS = 0%, CPS = 0). This result was interpreted as undetectable PD-L1 being a consequence of the tumor tissue disappearance subsequent to the patient’s achievement of pCR.

The classification of lung cancer has evolved from a purely histology-based approach to one incorporating molecular subtyping, driven by advances in our understanding of disease biology and sequencing technologies. Consequently, genetic profiles are increasingly becoming a prerequisite for treatment selection. As highlighted by Tan et al., genetic profiles serve as a cornerstone for precision medicine in lung cancer, informing not only first-line treatment strategies but also guiding re-biopsy and subsequent therapy adjustments following the development of resistance [30]. 

SCLC is characterized by significant intra-tumor heterogeneity (ITH), which profoundly influences treatment responses and prognosis. Supporting this, Zhang et al. identified ITH as a key driver of divergent therapeutic outcomes in SCLC, impacting both tumor biology and the state of the immune microenvironment. They developed a gene signature, ITHtyper, for prognostic stratification, which demonstrated a superior ability to predict immunotherapy response compared to PD-1/PD-L1 expression alone [31]. It is important to note, however, there are studies suggest the predictive value of SCLC transcriptional subtypes for prognosis may be limited, despite their observed heterogeneity .

Looking ahead, the integration of multi-omics technologies and innovative clinical trial designs holds promise for enabling more precise and dynamically adapted personalized therapy for lung cancer patients.

## Conclusion

This comparative study of two stage IIIB SCLC patients undergoing neoadjuvant chemoimmunotherapy elucidates critical interpatient heterogeneity in therapeutic responses and their underlying molecular determinants. Our findings demonstrate that SCLC-specific molecular features—including dynamic TMB trajectories, *MYC* amplification status, and distinct genomic alteration profiles—may critically influence immunotherapy sensitivity. Notably, the observed dissociation between PD-L1 expression levels and clinical outcomes challenges the biomarker paradigm established in NSCLC, underscoring the biological uniqueness of SCLC immune regulation. Future investigations should prioritize the integration of multi-omics analyses and optimization of neoadjuvant therapeutic strategies to improve clinical outcomes in SCLC patients.

## Data Availability

The raw data supporting the conclusions of this article will be made available by the authors, without undue reservation. The data can be obtained by contacting the corresponding author.

## Abbreviations

TMB: Tumor mutational burden
pCR: Pathological complete response
PD: Disease progression
SCLC: Small-cell lung cancer
ES-SCLC: Extensive-stage small-cell lung cancer
NSCLC: Non-small cell lung cancer
VAF: Variant allele frequence
ProGRP: Pro-gastrin-releasing peptide
CEA: Carcinoembryonic antigen
NSE: Neuron-specific enolase
VATS: Video-assisted thoracic surgery
TPS: Tumor proportion score
CPS: Combined positive score
CN: Copy number
ICI: Immune checkpoint inhibitor
TILs: Tumor-infiltrating lymphocytes
PFS: Progression-free survival
OS: Overall survival
ITH: Intra-tumor heterogeneity
